# Comparison of automated and retrospectively calculated estimated glomerular filtration rate in electronic health record data

**DOI:** 10.1186/s12882-018-1179-8

**Published:** 2018-12-28

**Authors:** Kristine E. Lynch, Ji won Chang, Michael E. Matheny, Alexander Goldfarb, Olga Efimova, Gregorio Coronado, Scott L. DuVall

**Affiliations:** 1 VA Informatics and Computing Infrastructure (VINCI), VA Salt Lake City Health Care System, Salt Lake City, UT USA; 2 Division of Epidemiology, Department of Internal Medicine, University of Utah, Salt Lake City, UT USA; 3 Geriatrics Research Education and Clinical Care Center, Tennessee Valley Healthcare System, Nashville, TN USA; 4 Department of Biomedical Informatics, Vanderbilt University Medical Center, Nashville, TN USA; 5 Beth Israel Deaconess Medical Center, Boston, MA USA; 6 Harvard Medical School, Boston, MA USA

## Abstract

**Background:**

Estimated glomerular filtration rate (eGFR) is the clinical standard for assessing kidney function and staging chronic kidney disease. Automated reporting of eGFR using the Modification of Diet in Renal Disease (MDRD) study equation was first implemented within the Department of Veterans Affairs (VA) in 2007 with staggered adoption across laboratories. When automated eGFR are not used or unavailable, values are retrospectively calculated using clinical and demographic data that are currently available in the electronic health record (EHR). Due to the dynamic nature of EHRs, current data may not always match past data. Whether and to what extent the practice of re-calculating eGFR on retrospective data differs from using the automated values is unknown.

**Methods:**

We assessed clinical data for patients enrolled in VA who had their first automated eGFR lab in 2013.We extracted the eGFR value, the corresponding serum creatinine value, and patient race, gender, and date of birth from the EHR. The MDRD equation was applied to retrospectively calculate eGFR. Stage of chronic kidney disease (CKD) was defined using both eGFR values. We used Bland–Altman plots and percent agreement to assess the difference between the automated and calculated values. We developed an algorithm to select the most parsimonious parameter set to explain the difference in values and used chart review on a small subsample of patients to determine if one approach more accurately describes the patient at the time of eGFR measurement.

**Results:**

We evaluated eGFR data pairs from 266,084 patients. Approximately 33.0% (*n* = 86,747) of eGFR values differed between automated and retrospectively calculated methods. The majority of discordant pairs were classified as the same CKD stage (*n* = 74,542, 85.93%). The Bland–Altman plot showed differences in the data pairs were centered near zero (mean difference: 0.8 mL/min/1.73m^2^) with 95% limits of agreement between − 6.4 and 8.0. A change in recorded age explained 95.6% (*n* = 78,903) of discordant values and 85.02% (*n* = 9371) of the discordant stages.

**Conclusions:**

Values of retrospectively calculated eGFR can differ from automated values, but do not always result in a significant classification change. In very large datasets these small differences could become significant.

## Background

Estimated glomerular filtration rate (eGFR) is a standard metric for assessing renal excretory function and staging chronic kidney disease (CKD) in routine clinical practice and is ubiquitously utilized in research settings. Clinically, eGFR can inform therapeutic strategy, disease prognosis, and is predictive of overall patient survival [1–8]. From a research standpoint, eGFR can be used as selection criteria for entry into observational cohorts [9–12] or clinical trials [13–17], as well as be an exposure, outcome, or covariate of interest [12, 18–22]. The National Kidney Foundation Disease Outcomes Quality Initiative (K-DOQI) and the National Kidney Disease Education Program (NKDEP) recommend that in absence of direct measurement of renal excretory function, eGFR can be calculated from prediction equations based on factors commonly found in the electronic health record (EHR) [1, 4]. One equation is the Modification of Diet in Renal Disease (MDRD) study equation, which derives eGFR from patients’ age, race, and serum creatinine (S_cr_). This is an older equation that is being slowly phased out in favor of the CKD Epidemiology Collaboration (CKD-EPI) equation, [23] however, real-time calculations in EHRs still use the MDRD equation as do many research groups [9, 19, 20, 24, 25].

Since 2002 the K-DOQI, NKDEP and International Society for Nephrology have encouraged laboratories to automate eGFR reporting [1, 2, 4]. That is, to implement software in clinical laboratories that automatically calculates and reports eGFR in real-time alongside each corresponding S_cr_ value. Although recommended, universal adoption of the automated process has been varied with gradual and incomplete implementation across laboratories in the United States (US) [26, 27]. The NKDEP and the College of American Pathologists’ (CAP) annual surveys determined that only 40–50% of US laboratories used automated eGFR in 2007 and most independent laboratories reported eGFR only when specifically requested by a clinician [26, 27]. Integrated health care systems tend to have higher rates of implementation than independent laboratories. For example, the Alberta Health Services in Alberta, Canada, had 100% implementation in 2004, [28, 29] and the Department of Veterans Affairs (VA), one of the largest integrated health care system in the US, had approximately 68% of its laboratories using automated reporting in 2007 [30].

The VA uses a system-wide EHR, known as the VistA to store patient information dating back to the 1990s. In 2004, the VA National Pathology and Laboratory Service created an eGFR software patch for VistA that enabled each laboratory’s information technology system to automatically calculate eGFR using the isotopic dilution mass spectrometry (IDMS)-traceable MDRD equation and report eGFR values with S_cr_ results [30, 31]. Because each VA laboratory had to independently download the patch, integration was staggered across time.

In research settings, even when automated eGFR is available, it may not be utilized. For one, researchers may not always rely on the automated values and recalculate eGFR themselves using the equation parameters. Second, automated values may not be available for every patient in a cohort during a pre-specified fixed time window (e.g., one year prior to an index date). Limiting the study population to CKD patients with non-missing automated eGFR may result in suboptimal sample sizes and potential loss of statistical power. Alternatively, a researcher can impute missing eGFR values using the MDRD equation. Both of these self-calculation scenarios require that age, gender, race, and S_cr_ are available to the researcher and run on the assumption that the values of these four parameters at the time of study execution accurately reflect the patients’ values at the time point of interest. In some cases, the interval between the time point of interest and study execution date can be decades. Given the dynamic nature of the EHR, the data the researcher uses to impute eGFR values (referred to hereinafter as retrospectively calculated eGFR) may not always match past data in which the imputed value was meant to represent. As such, the retrospectively calculated eGFR value may differ from the automated eGFR value. How often and to what extent the two values can disagree is unknown. The goal of this study therefore was twofold: 1) to quantify the agreement between automated and retrospectively calculated eGFR and 2) determine which equation parameter(s) explain any observed disagreement.

## Methods

### Data source

To address the research question, we used data available in the VA’s Observational Medical Outcomes Partnership (OMOP) common data model database [32]. VA-OMOP is a transformation of VA’s Corporate Data Warehouse’s (CDW) to the OMOP common data model. The CDW is a nationwide repository, storing all patient-level data recorded from the VA system-wide EHR. It contains historical data dating back to October 1, 1999, including demographic, visit, provider, inpatient and outpatient diagnoses, medication, and lab data [33–35]. Data from laboratories that implemented the eGFR software patch are fed into CDW nightly similar to other laboratory data.

### Cohort selection and variable creation

We identified patients enrolled in the VA who had their first automated eGFR lab between January 1, 2013 and December 31, 2014. We chose this time period, ten years after the VA first implemented the eGFR software patch, to provide time for clinical practice to potentially adopt the patch and to ensure broad geographical representation of VA laboratories in our study. We identified laboratory values of automated eGFR produced from the patch by a combination of string search in lab test names and verified with clinical review. We extracted the first eGFR value recorded in 2013 and rounded to the nearest tenth decimal place. We then extracted the S_cr_ value (measured enzymatically) that occurred on the same date as the eGFR lab. Lab values of S_cr_ were pulled using Logical Observation Identifier Names and Codes (LOINC) (35203–1, 77,140–2, 21,232–4, 2160–0, 38,483–4, 59,826–8, 14,682–9). In addition to S_cr_, patient race, gender, and age at S_cr_ (using DOB) documented in the medical record at time of query (September 1, 2017) were applied to the IDMS MDRD equation ((175 × (S_cr_)-1.154 × (Age)-0.203 × (0.742 if female) × (1.212 if African American)), [36] and eGFR was retrospectively calculated and rounded to the nearest tenth decimal place. For demonstration purposes only and to assess whether differences between the two eGFR values crossed clinically significant boundaries, patients were categorized into CKD groupings according to both automated and retrospectively calculated eGFR (eGFR + 90; eGFR 89–60; Stage 3a = 59–45; Stage 3b = 44–30; Stage 4 = 29–15; Stage 5 = < 15). In clinical practice, Stage 1 and Stage 2 CKD are only diagnosable in the setting of other conditions (e.g., proteinuria, history of kidney transplantation, pathological abnormalities), whereas Stage 3+ can be staged according to eGFR alone [37, 38].

Because demographic data were needed to retrospectively calculate eGFR, any patient missing data on race, DOB, or gender was excluded from subsequent analyses.

### Data analysis

Population demographics were assessed using descriptive statistics. Continuous variables are presented as means and standard deviations (SD) and categorical variables as frequencies and percentages.

We used Bland–Altman plots [39] to assess the difference between retrospectively calculated eGFR values and automated eGFR values. We determined each patient’s stage of CKD according to both automated and retrospectively calculated eGFR (mL/min/1.73 m^2^) and assessed differences in stage assignment using percent agreement and the Kappa (ĸ) coefficient.

**Fig. 1:**
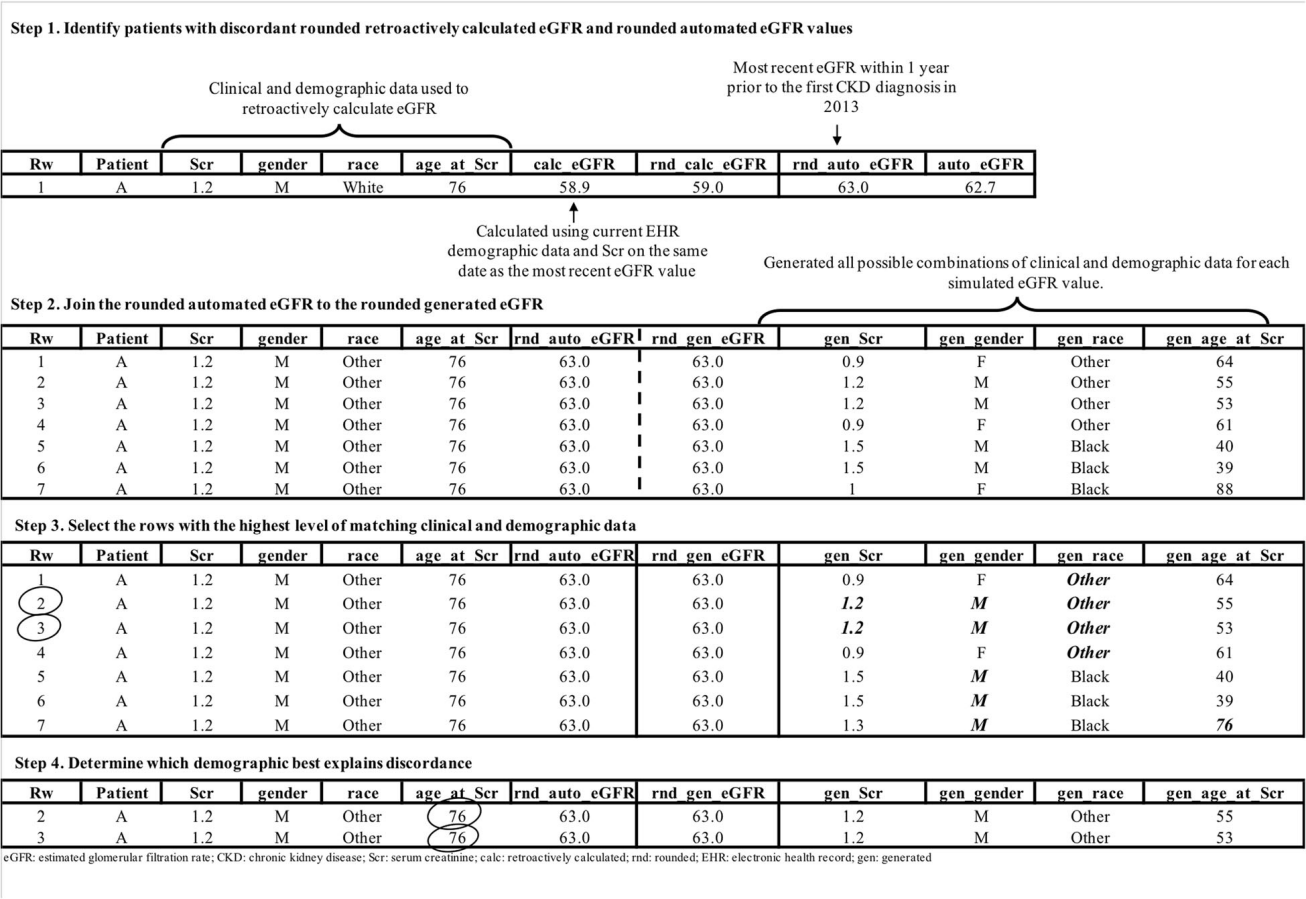
Process for determining source of discordant eGFR values

We implemented a four-step process to determine if the difference between values could be attributed to a change in one or more MDRD formula parameters in a patient’s EHR over time (Fig. 1). Data presented in Fig. 1 are modeled on actual instances we found in patient records, but do not contain any actual patient data. First, to avoid including pairs that were discordant because of differences in input parameter rounding (i.e., age and Scr), we conservatively selected all patients whose rounded automated eGFR and rounded retrospectively calculated eGFR pairs were not equal (Step 1). In Step 1 for example, the calculated eGFR was rounded from 58.9 to 59.0 and the automated eGFR from 62.7 to 63. We considered this pair discordant and proceeded to Step 2. Had the automated eGFR been 58.9 instead of 62.7 we would have considered both the automated and calculated eGFR to be 59.0 and a concordant pair. We generated all plausible eGFR values rounded to the nearest tenth decimal place resulting in a range from 1.0 to 251.0 mL/min/1.73 m^2^. Using the MDRD equation, we iteratively determined every combination of age (18–120 years of age), race (black, non-black), S_cr_ (0.10–30.0 mg/dL) and gender (male, female) that could have yielded each generated eGFR value. We then joined the automated value to the generated eGFR value and compared the generated demographics and S_cr_ to the current demographics and S_cr_ used to retrospectively calculate eGFR (Step 2). For each pairwise comparison, we assessed how many variables matched between the generated and current demographics and S_cr_. We hypothesized a priori, that although possible, it was very unlikely that all equation variables changed in the EHR since the date eGFR was recorded, and further analysis was limited to comparisons with the highest level of matching information (Step 3). From there, we were able to identify the possible equation parameter(s) that explained the discordance (Step 4). In the example presented in Fig. 1 the difference between automated and retrospectively calculated eGFR was explained by age. Frequencies and percentages for each explanatory parameter(s) were separately calculated for discordant eGFR values and CKD stages. We assessed a subset of pairs that were discordant by both eGFR value and CKD stage and identified the possible equation parameter(s) that could explain the disagreement.

Lastly, medical record abstraction was performed by two trained clinical annotators on 20 discordant eGFR dyads to determine if one of the methods was more prone to error than the other. We randomly selected 5 pairs with discordant CKD stage from each explanatory element grouping- age, race, gender, and S_cr_. We reviewed medical text notes from the time period surrounding the date stamp of the eGFR lab to determine values of age, race, gender, and S_cr_ at the time of automated eGFR. For demographic variables, we then examined all note types surrounding the index date to determine whether the retrospectively calculated demographic value or the automated demographic value more accurately described patient. For example, if race explained the difference between two values and the retrospectively calculated race value did not match the race value in the medical note on the date of the automated eGFR lab we concluded that our algorithm correctly identified the explanatory parameter. If we found the majority of race mentions in the entirety of the medical record to match the retrospectively calculated race we concluded that the retrospectively calculated eGFR was the most appropriate value. Conversely, if we found the opposite to be true, we concluded that the automated eGFR was the most appropriate value. Lastly if the two values differed, and we could not find any evidence in the medical notes to support the explanatory results, the most appropriate value was undeterminable.

Analyses were performed using SAS software, version 9.4 (SAS Institute, Inc. Cary, NC) and R version 3.2.2 (R Foundation for Statistical Computing, Vienna, Austria).

## Results

Between January 1, 2013 and December 31, 2014, we identified 307,292 patients with their first eGFR lab in 2013. All 307,292 patients had a S_cr_ lab value on the same day as the eGFR lab, however we excluded 2540 patients whose S_cr_ was part of the comprehensive metabolic panel leaving 304,752 patients. These S_cr_ values were available as unstructured text, but to abstract them through chart review was outside the scope of this study. The non-panel S_cr_ test results were available as structured data. Lastly, we excluded 14,623 patients without a race documented in the medical record at time of query (September 1, 2017). The final analytic cohort consisted of 266,084 patients. The mean age of our overall population was 54.87 (standard deviation = 27). The majority of patients were male (90.33%) and identified as white (79.71%) (Table 1).

**Table 1:** Characteristics of the study cohort

	Overall *N* = 266,084		Discordant eGFR values *N* = 86,747		Concordant eGFR values *N* = 179,337		
Characteristic	Mean	SD	Mean	SD	Mean	SD	*p*-value
Age	54.87	27.00	51.83	18.36	56.33	17.90	< 0.05
Scr	1.04	0.51	1.02	0.39	1.05	0.55	0.74
Retroactively calculated eGFR	83.16	28	84.88	23.04	82.33	22.9	< 0.05
Automated eGFR	84.00	27	87.25	23.48	82.41	22.94	< 0.05
Gender	N	%	N	%	N	%	< 0.05
Female	25,725	9.67	9933	11.45	15,792	8.81	
Male	240,359	90.33	76,814	88.55	163,545	91.19	
Race binary							< 0.05
Black	46,873	17.62	19,380	22.34	27,493	15.33	
Other	219,211	82.38	67,367	77.66	151,844	84.67	
Race							< 0.01
Am. Indian	3068	1.15	1034	1.19	2034	1.13	
Asian	4058	1.53	1310	1.51	2748	1.53	
Black	46,873	17.62	19,380	22.34	27,493	15.33	
White	212,085	79.71	65,023	74.96	147,062	82.00	

*SD* standard deviation, *Scr*: serum creatinine, *p*-values generated from independent t-tests and chi square tests

**Fig. 2:**
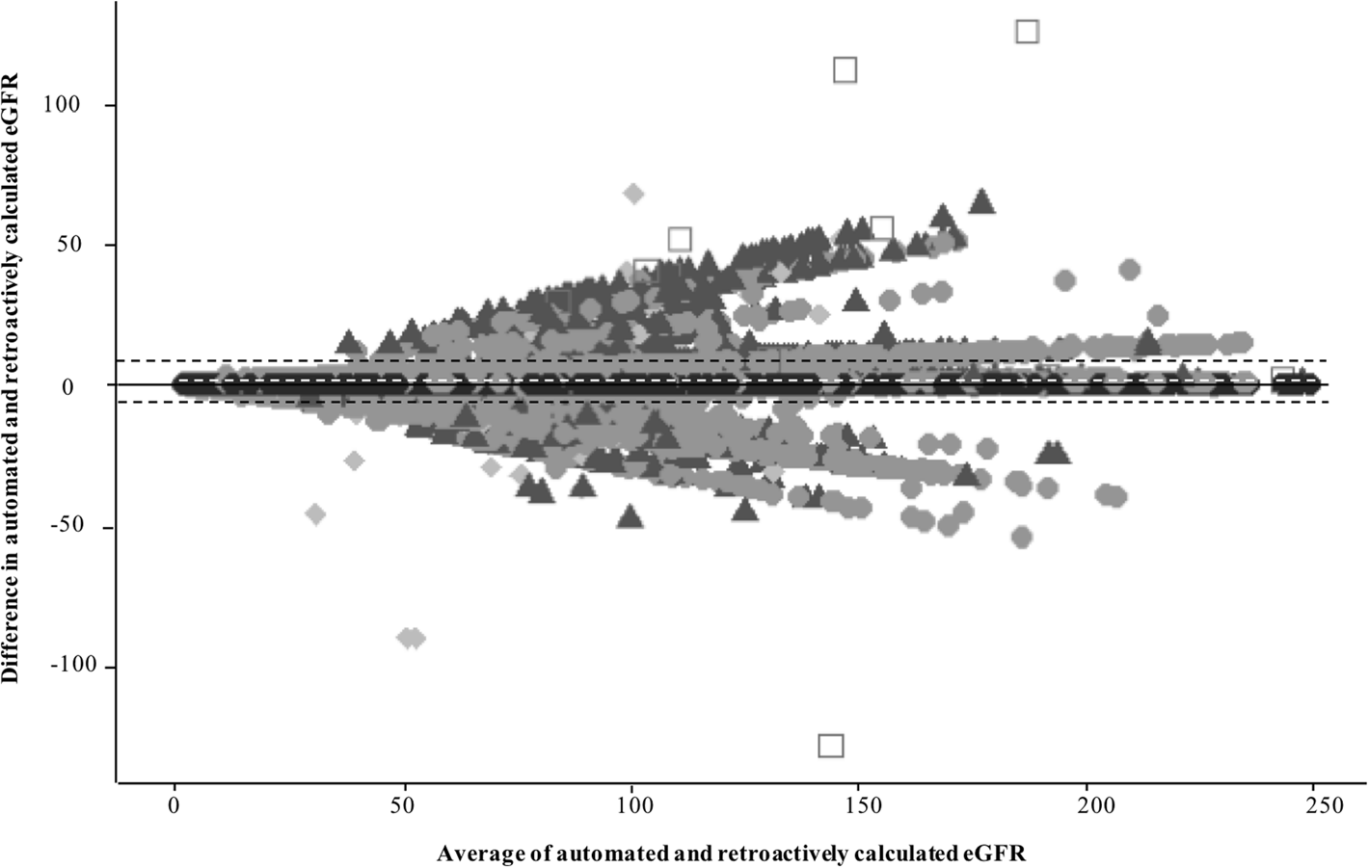
Bland Altman Plot of automated and retrospectively calculated eGFR. Black solid line is drawn at the zero difference in automated and retroactively calculated eGFR. White dashed line (0.8) represents the average difference of automated and retroactively calculated eGFR. Black dashed lines (8.0, −6.4) indicate the 95% limits or +/− 2 standard deviations from the average difference of automated and retroactively calculated eGFR. The average automated eGFR was 83.99 and for retroactively calculated eGFR 83.16. The shapes refer to explanatory factors. Black circles = match, grey circles = 1 demographic, white rectangle = 1 demographic and 1 clinical, triangle = 2 demographics, diamond = 1 clinical

The mean automated eGFR was 84.00 mL/min/1.73m^2^ and 83.16 mL/min/1.73m^2^ for retrospectively calculated eGFR, ranging 2 to 250 mL/min/1.73m^2^ across both methods. Approximately 33.0% (*n* = 86,747) of patient’s eGFR values differed between automated and retrospectively calculated methods. Patients with discordant eGFR values were slightly younger and comprised more female and black patients than those with concordant eGFR values (Table 1). The Bland-Altman plot showed differences in the data pairs were centered near zero (mean difference: 0.8 mL/min/1.73m^2^) with 95% limits of agreement between − 6.4 and 8.0 (Fig. 2). The majority of the 86,747 pairs with discordant eGFR values were classified as the same CKD stage (*n* = 74,542, 85.93%). The mean difference between pairs with discordant values and CKD stage was 7.56 mL/min/1.73m^2^ and 4.34 mL/min/1.73m^2^ for pairs with discordant values and concordant CKD stage.

According to both retrospectively calculated eGFR and automated eGFR, the most common stage of CKD was eGFR 89–60 (50.65 and 49.13%, respectively) followed closely by eGFR 90+ (36.29 and 38.07%). The least common stage of CKD was Stage 5 as defined by both methods (0.33%). Overall CKD stage discordance between the two methods was 4.60% (*n* = 12,205). Patients with eGFR 89–60 according to retrospectively calculated eGFR and eGFR 90+ according automated eGFR represented the most common discordant pairs (*n* = 6495, 2.44%). Extreme stage discordance was rare. For example, only 2 patients were identified as eGFR 90+ by retrospectively calculated eGFR and Stage 5 by automated eGFR. The overall Kappa of 92.41 indicates almost perfect agreement between the two methods [40]. The complete agreement matrix is presented in Table 2.

**Table 2:** Agreement matrix of CKD stage by retrospectively calculated and automated eGFR values

CKD stage by automated eGFR, *n* (%)	CKD stage by retrospectively calculated eGFR, n (%)						Total
	eGFR + 90	eGFR 89–60	Stage 3a (59–45)	Stage 3b (44–30)	Stage 4 (29–15)	Stage 5 (< 15)	
90+	94,809 (35.63)	6495 (2.44)	0 (0)	0 (0)	0 (0)	0 (0)	101,304 (38.07)
89–60	1758 (0.66)	127,020 (47.74)	1935 (0.73)	1 (0)	0 (0)	0 (0)	130,714 (49.13)
Stage 3a	1 (0)	1264 (0.48)	22,061 (8.29)	509 (0.19)	0 (0)	0 (0)	23,835 (8.96)
Stage 3b	0 (0)	0 (0)	118 (0.04)	7144 (2.68)	74 (0.03)	0 (0)	7336 (2.76)
Stage 4	0 (0)	0 (0)	1 (0)	27 (0.01)	1986 (0.75)	11 (0)	2025 (0.76)
Stage 5	2 (0)	0 (0)	1 (0)	0 (0)	8 (0)	859 (0.32)	870 (0.33)
Total	96,570 (36.29)	134,779 (50.65)	24,116 (9.06)	7681 (2.89)	2068 (0.78)	870 (0.33)	

Kappa = 92.41 (92.27–92.54)

Demographics were the equation parameters most likely to explain discordant eGFR values as well as CKD stage. A change in age, race, gender, or combination explained 99.2% (*n* = 86,055) of the value differences and 97.7% (*n* = 11,931) of the stage differences. A change in recorded age explained 95.6% (*n* = 78,903) of discordant values and 85.02% (*n* = 9371) of the discordant stages with the absolute median difference in age for these pairs being 18.0 and the median difference in eGFR being 4.00 mL/min/1.73m^2^. Race alone explained 2.6% and S_cr_ explained 0.6% of conflicting values with the median difference in values being 12.00 and 15.00 mL/min/1.73m^2^, respectively. The absolute median difference in S_cr_ values was 0.20 mg/dL. Race explained 9.88% and S_cr_ explained 2.18% of conflicting CKD stages with the median difference in values being 16.00 and 12.00 mL/min/1.73m^2^, respectively. For the pairs that were discordant both in value and CKD stage, race alone explained more of the difference than for pairs discordant in value but concordant in stage (9.88% vs. 1.49%, respectively). See Table 3 for complete results.

**Table 3:** Explanatory parameters of value and stage discordance

Equation parameter(s)	Median of absolute difference	Number of pairs	%
Discordant eGFR value			
Any	4.0	86,747	100.00
1 clinical	12.0	484	0.56
1 demographic 1 clinical	7.0	208	0.24
2 demographics	8.0	3562	4.11
1 demographic	4.0	82,493	95.10
Race	15.0	2151	2.61
Gender	29.0	151	0.18
Age	4.0	78,903	95.65
Gender or age	29.0	10	0.01
Race or gender	1.0	6	0.01
Race or age	16.0	1265	1.53
Race, gender, or age	1.0	7	0.01
Discordant eGFR value and stage			
Any	4.0	12,205	100.00
1 clinical	12.0	266	2.18
1 demographic 1 clinical	34.5	8	0.07
2 demographics	12.0	909	7.45
1 demographic	6.0	11,022	90.31
Race	16.0	1089	9.88
Gender	25.0	73	0.66
Age	5.0	9371	85.02
Gender or age	28.0	6	0.05
Race or gender	0.0	0	0.00
Race or age	17.0	483	4.38
Race, gender, or age	NA	0	0.00
Discordant eGFR value only			
Any	6.0	74,542	100.00
1 clinical	12.0	218	0.29
1 demographic 1 clinical	7.0	200	0.27
2 demographics	7.0	2653	3.56
1 demographic	4.0	71,471	95.88
Race	14.0	1062	1.49
Gender	35.0	78	0.11
Age	4.0	69,532	97.29
Gender or age	34.0	4	0.01
Race or gender	1.0	6	0.01
Race or age	15.0	782	1.09
Race, gender, or age	1.0	7	0.01

*eGFR* estimated glomerular filtration rate, Clinical parameter: serum creatinine; Demographic parameters: age, race, gender

Our chart review process revealed that for all 5 discordant pairs explained by a change in S_cr_, the current S_cr_ was the actual value and the retrospectively calculated eGFR was the preferred method. The notes specifically stated that the original S_cr_ value was erroneous and the incorrect value was changed to the new value. For age and race, we found the preferred method as automated for 2 pairs, retrospectively calculated for 2 pairs and undeterminable for 1 pair. For gender, we found the preferred method as retrospectively calculated for 3 pairs and automated for 2 pairs.

## Discussion

We sought to examine whether and to what extent retrospectively calculated eGFR can differ from automated eGFR values. Results illustrated that changes in MDRD equation parameters over time are fairly common in EHR data which can lead to changes in eGFR values and sometimes even changes to CKD stage classification. We found 32.6% discordance between the retrospectively calculated and automated values and approximately 5% of these differences were large enough to result in CKD stage discordance.

Changes in demographic variables largely explained incongruences between retrospectively calculated and automated eGFR with age being the predominate explanatory factor. Changes in S_cr_ accounted for the least number of differences. Our findings make sense intuitively. Age is calculated from date of birth. Due to the self-reporting nature of date of birth at each clinical encounter there is more opportunity for error than there is for a laboratory value. A patient’s day, month, year, or combination can be misreported because of recording or processing errors [41]. Race, though similarly self-reported, explained far less discordance than age. The only race change that could affect eGFR is black to non-black (and vice versa). Any change in race assignment over time that was not a change from black to non-black or from non-black to black would not change eGFR. For example, a white female who is 60-year-old with S_cr_ of 1.1 mg/dL would have the same eGFR as an Asian, Pacific Islander, or Native American female with the same age and S_cr_.

We found the most common stage discordance between eGFR 90+ and eGFR 89–60 and only 6 instances of stage discordance crossed clinically important boundaries (e.g., eGFR 90+ and Stage 5 or Stage 3a and Stage 4). In all 6 instances either S_cr_ alone or S_cr_ and one demographic accounted for the difference. Since eGFR and S_cr_ are stored as separate data elements, a change to a S_cr_ value does not automatically trigger a change to the corresponding eGFR lab value. After reviewing a small sample of clinical notes, it appears that changes in S_cr_ occur to replace erroneous lab values and historical records are not preserved as structured data. For these instances, the retrospectively calculated eGFR may be the preferred approach to be adopted. However, changes in S_cr_ accounted for < 1% of value discordance and < 3% of stage discordance. There appeared to be no systematic explanations for race, gender, or age changes. In other words, sometimes the automated approach appeared to better reflect the patient’s eGFR and sometimes the retrospectively calculated approach was better. For example, one patient was recorded as white in the note corresponding to the automated eGFR lab, but was recorded as black in every previous and subsequent note as well as in the current structured race field. For this patient, we considered the retrospectively calculated eGFR the preferred approach. Another patient was recorded as male in the note corresponding to the eGFR lab and in the majority of subsequent notes, but the structured gender field was set as female. The most recent medical notes specified that the patient now prefers female pronouns. For this patient, we concluded that the automated eGFR was the preferred approach. With the absence of a gold-standard comparison (e.g., direct measurement of GFR) to determine whether one method was a better reflection of patients’ true GFR, we relied on chart review to determine whether the retrospectively calculated or the automated equation parameter values more accurately described the patient at the time point of interest. A larger chart review study may be warranted to determine if one method is statistically more accurate than the other.

To our knowledge, this is the first study to assess differences between automated and retrospectively calculated eGFR although both are used in research [42]. The VA was an ideal setting for this study as CKD is highly prevalent among Veterans, [42] providing a large national sample. The VA EHR is dynamic, updating and adding data including over 1 million medical notes and reports each night, however findings from this research are generalizable to any dynamic EHR data. Electronic health records can have varying methods of maintaining historical data. Some have destructive replacement practices; meaning when a new entry is made the previous entry for that instance is deleted and replaced. Our findings may also have utility for other measures that are can be similarly retrospectively calculated using EHR data such as body mass index (kilogram/meter^2^), urine albumin-to-creatinine ratio (urine albumin/ urine creatinine), etc.

In the VA, automated eGFR is calculated using the MDRD equation. There are limitations to this equation and alternative approaches such as the CKD-EPI equation have been proposed. It was designed to match the accuracy of the MDRD equation at GFR < 60 mL/min/1.73m^2^ and offers greater accuracy at higher GFR, minimizing the over-diagnosis of CKD [38]. The improved accuracy of the CKD-EPI equation may result in the CKD-EPI replacing the MDRD study equation as the preferred tool for CKD screening and risk stratification [23]. Notwithstanding, health systems or researchers that utilize the CKD-EPI equation are prone to the same issues discussed in the present study as it uses the same demographic and clinical variables as the MDRD eq. (141 × min (S_cr_/κ,1)^α^ × max (S_cr_/κ, 1)^-1.209^ × 0.993^Age^ × 1.018 [if female] × 1.159 [if African American]).

Although we observed 33% of differing eGFR values, the differences between values were small and likely not to have much impact in terms of bias on risk estimates such as risk ratios or hazard ratios. However, measurement error has the potential to influence prediction models. Specifically, random error can create instability and error rates in individual predictions and problems with calibration (i.e. agreement between observed and predicted rates) [43]. Aside from the eGFR pairs explained by changes in S_cr_, it was not apparently clear whether one approach was better than the other. However, the retrospectively calculated eGFR confers some benefits for researchers, as it can significantly improve data completeness and allow for the application of equations not automated through the health system’s EHR (e.g., CKD-EPI in VA). Further, the challenge of automated eGFR is when the preferred method changes over time. Retrospective calculations can use the latest or most preferred equation for all previous values irrespective of the automated equation.

## Conclusions

The widespread adoption of EHRs provides much opportunity for secondary-use of clinical data for nephrology research purposes. Study design specifications can warrant retrospective calculation of eGFR using stored demographic and clinical values. However, due to the dynamic nature of most EHRs, record of race, gender, age, and even serum creatine values can change over time and impact eGFR calculated values. In our assessment we found differences between retrospectively calculated eGFR and automated values are common and can result in differences in disease classification. In very large datasets or prediction studies these differences could become significant. It is important to consider the validity of variables used to calculate eGFR when utilizing EHR data.

## Abbreviations

CAP: College of American Pathologists
CDW: Corporate Data Warehouse
CKD: Chronic Kidney Disease
CKD-EPI: Chronic Kidney Disease Epidemiology Collaboration
eGFR: Estimated Glomerular Filtration Rate
EHR: Electronic Medical Record
K-DOQI: National Kidney Foundation Disease Outcomes Quality Initiative
LOINC: Logical Observation Identifier Names and Codes
MDRD: Modification of Diet in Renal Disease
NKDEP: National Kidney Disease Education Program
OMOP: Observational Medical Outcomes Partnership
S_cr_: Serum Creatinine
SD: Standard Deviation
VA: Department of Veterans Affairs
